# Enhancing multiclass plant disease classification using GAN-boosted vision transformer with XAI insights

**DOI:** 10.3389/fpls.2025.1649399

**Published:** 2026-01-09

**Authors:** Felicita S. A. M., Kavitha B. R.

**Affiliations:** School of Computer Science Engineering and Information Systems, Vellore Institute of Technology, Vellore, Tamil Nadu, India

**Keywords:** rice disease detection, vision transformer, generative adversarial networks, explainable AI, deep learning, class imbalance

## Abstract

**Introduction:**

Agriculture is one of the major backbones of the Indian economy, where rice is the most prominent staple crop across the country. However, rice production has been significantly affected due to the occurrence of various plant diseases. Deep learning and machine learning have emerged as powerful solutions for computer vision-based problems.

**Methods:**

This work identifies some of the key diseases and addresses these prominent ones using a state-of-the-art deep learning model. It proposes a novel multiclass rice leaf disease recognition model named GRG-ViT, which integrates Vision Transformer (ViT), Generative Artificial Intelligence (GenAI), and Explainable Artificial Intelligence (XAI) techniques for better outcomes. The Vision Transformer-based framework is designed to capture long-range spatial dependencies in leaf images, which enhances the model’s ability to identify the subtle disease patterns. Since the dataset portrayed considerable class imbalance, a GenAI-based synthetic data generation approach is equipped in this model to create balanced training samples, which in turn improves the model’s robustness. This model also proposes a hybrid Rectified Linear Unit (ReLU)–Gaussian Error Linear Unit (GELU)-based activation mechanism to attain effective feature representation.

**Results and discussion:**

The obtained experimental results exhibit that the proposed GRG-ViT model reaches close to an overall accuracy of 96%, which outperforms conventional approaches. The incorporation of XAI methods like Gradient-weighted Class Activation Mapping (Grad-CAM) provides both interpretability and transparency by emphasizing the regions impacting the model’s actions. This research showcases the blended power of ViT, GenAI, and XAI in producing reliable and high-performing results for rice disease detection in precision agriculture.

## 1 Introduction

Agriculture is the fundamental resource for feeding human beings and faces huge challenges in recent days in the form of climate change, water scarcity, soil degradation, pests, and diseases. These challenges pose a significant threat to food security, especially considering the growing population. That is why, as one of the significant steps to address this, the United Nations has announced “Zero Hunger” as the second most important Sustainable Development Goal. Considering the above facts, among the prominent threats to agricultural production, plant infections and diseases are chosen as the objective of this work, which aims to ensure the quick and early detection of diseases. This will help address one of the pressing issues that agriculturists are facing in recent days.

It is estimated that major crops like rice, wheat, and potato face 10% to 40% losses in production due to leaf diseases (Parez et al., 2023). As these pose a big threat to major crops, they necessitate frequent inspection of each crop, which is a very time-consuming process, especially in a world where there is a huge shortage of agriculture-based labor. Therefore, it is necessary to automate this process to help the agricultural community with the latest developments in computing. Such automatic identification not only saves time but also ensures significant improvements in production, which subsequently addresses issues of income loss that naturally persist among farmers. In this work, we have considered the rice crop, as it contributes approximately 25% of the overall agricultural production in India and 25% of rice production worldwide.

Machine learning and deep learning are two advanced technologies under artificial intelligence that address most of these growing issues. These advanced algorithms have the capability to handle huge amounts of data being generated. When leaf diseases need to be identified properly, the appropriate classification of the disease becomes helpful in treating it in a timely manner. Many prominent algorithms, like Convolutional Neural Network (CNN), have been very successful in solving issues related to computer vision, and researchers in the past have also achieved good accuracy in predicting plant diseases (Borhani et al., 2022). In many cases in recent times, some of the tiny features present in the leaf mislead the disease prediction process manually, and it requires an advanced methodology to accurately predict the exact disease. It poses challenges in identification and may also go unnoticed sometimes.

Rice is the primary food source for the entire South Indian region. In the global context, close to 35% of the entire population consumes rice (Zhou et al., 2023). Therefore, the selection of this particular crop in our work has an appropriate justification, as it is more relevant among all major crops. The identification of crop diseases requires leaf images, and handling image-based data demands strong deep learning-based approaches. Even though CNN-based algorithms are highly successful in solving image-based classification or identification tasks, they face some key challenges and limitations. These include data dependency, high computational complexity and memory requirements, and difficulty in handling imbalanced data.

Vision Transformer (ViT), which was introduced a couple of years ago, has shown good progress in handling image-based data. It especially addresses some of the key limitations of CNN-based approaches. Among the key highlights of this approach are its scalable nature, which fits any size of data. Processing through a sequence of patches ensures that long-range dependency is maintained, which is another key advantage of this approach. Its ability to work in patches enables its adaptability to different image resolutions or multimodal data. Its simpler architecture is also a key advantage, as it greatly reduces computational complexity when compared to CNN. Overall, transformers have proven to be better at capturing contextual relationships across images.

A Generative Adversarial Network (GAN) is a generative artificial intelligence (AI) approach that helps to address image imbalance issues. It has the ability to generate realistic and diverse synthetic images and thus serves multiple applications (Kas et al., 2024) like super-resolution, anomaly detection, image synthesis and editing, image restoration, and style transfer (Zhang et al., 2025). Its image augmentation capability helps in solving many problems where class imbalance persists. In this work, we are utilizing this approach to balance our data samples. Deep learning-based models are generally termed black boxes, and we may not always be able to interpret the results. Explainable Artificial Intelligence (XAI) techniques have brought better transparency to these models, especially in terms of result interpretation (Budhkar et al., 2025). Gradient-weighted Class Activation Mapping (Grad-CAM), LIME, and SHAP are some of the prominent XAI approaches that help in this interpretation process. Grad-CAM helps in highlighting the key regions, whereas the attention mechanism, which is exclusively available for ViT, helps visualize the image patches.

With the key advantages that Vision Transformer offers, in our work, we are developing a hybrid model with the help of Vision Transformer and Conditional GAN to address the challenges of this multiclass problem by enhancing efficiency in identifying plant diseases. Especially in handling multiclass problems, normal deep learning approaches suffer in achieving class-based accuracy, and our approach addresses this limitation by achieving greater accuracy compared to other prominent models. The different classes of rice diseases, namely, black stem borer, white stem borer, yellow stem borer, brown spot, hispa, and bacterial leaf blight (BLB), are considered in this work based on the severity of the disease. The proposed model achieves better accuracy compared to other existing models, and the outcome of this model is compared in terms of different metrics.

The key objectives and contributions of this work are given as follows:

- Following the success of Transformers in vision-based problems, we are proposing a model based on Vision Transformer for rice leaf disease detection, which achieves 96% overall accuracy.
- Our model addresses one of the key complexity issues of multiclass classification, and it classifies all the chosen seven classes efficiently.
- In our model, to synthesize the leaf images, we have utilized Generative Artificial Intelligence (GenAI)-powered data augmentation, where a Conditional GAN has been used to generate realistic leaf images, which helped in balancing the dataset.
- This model overcomes the shortcomings of CNN-based models in terms of prediction accuracy as well as computational complexity, which is demonstrated in the result analysis part.
- Finally, the model transparency is illustrated using XAI-based techniques. This integration enhances our model’s credibility and trustworthiness. This part also enables a possible deployment for real-time agricultural applications.

The rest of the paper is organized as follows: the second section discusses the recent literature, the third section depicts the methodologies used with the proposed model, the fourth section explains the various result analysis parts, and the final section illustrates the conclusion of this work.

## 2 Related works

Plant disease, a deadly cause of crop failure, leads to huge losses for the farmers who completely depend on plant yield for their daily needs. To aid them, many researchers have developed new prediction and classification-based models with the help of AI-based learning methods like machine learning, deep learning, and transfer learning. Many researchers have already suggested different classification techniques for precision agriculture using the abovementioned algorithms (Chakrabarty et al., 2024; Reis and Turk, 2024). Some of the key literature considered in this work is discussed in this part of the paper.

A novel Crop Leaf GAN (CLGAN) for various maize leaf disease classifications was proposed by certain researchers. This work aimed to increase the accuracy and optimize the loss functions with minimal parameters. The GAN was built with an encoder and decoder for the generator and discriminator to reduce the vanishing gradient problem (Sharma et al., 2024). Min Peng et al. designed a Dimension Reduction Fuzzy Graph Network (DRFG), a fusion approach that combines the fuzzy technique DR with 3D-CNN and GAT for the analysis and classification of hyperspectral images (Peng et al., 2024).

Another work proposed an IoT- and deep learning-based model for weather forecasting, field monitoring, and disease classification for apple leaves. The Gated Recurrent Unit (GRU) was used for weather forecasting, and ResNet-50 was used for disease prediction and was automated with the help of sensors to support precision agriculture (Akilan and Baalamurugan, 2024). In order to achieve precise classification and detection of leaf diseases in tomato plants, the researchers incorporated the detection mechanisms SimAM and DAiAM over a YOLOv7 network. The images were segmented using the SIFT technique to extract crucial features, and max-pooling was used to reduce information loss. This target detection model predicted seven types of leaf diseases (Umar et al., 2024).

Roopali Dogra et al. proposed a deep learning-based model to precisely detect a particular rice leaf disease called brown spot using CNN-VGG19 integrated with transfer learning. The leaf images were collected from Jhansla village in Punjab, and the process involved image acquisition, feature extraction, image classification using max-pooling with different activation functions, and finally prediction with 93% accuracy. However, this work considered only one disease, brown spot (Maurício et al., 2023). In another study, the researchers suggested an integrated CNN-BiGRU model to classify four different rice leaf diseases. They achieved this by extending the inception module functionalities and implementing a residual mechanism. The Convolutional Block Attention Module (CBAM) was combined with CNN to precisely extract features, and CNN-BiGRU recognized the relationships between images to classify their respective classes (Xin et al., 2022).

Later, with the advancements of transformers in image classification, many researchers started working in this direction. José Maurício et al. reviewed several recent papers to identify the best state-of-the-art model between Vision Transformer and Convolutional Neural Network to determine which performs better on the image classification problem. Vision Transformer, with its multi-head attention mechanism, outperforms CNN due to its long-range dependencies and capability of adapting to different input sizes and noisy images (Abbas et al., 2021). In medical imaging, several AI-based algorithms have recently been introduced. Among them, one such work used a Vision Transformer to perform conventional classification of skin cancer images. The self-attention mechanism of Vision Transformer helped extract the important features of the image while excluding noise-producing features, which in turn helps in the early prediction of cancer cells (Liu et al., 2025). A hierarchical approach for plant disease detection on the PlantVillage dataset was suggested by researchers. For training and feature extraction, they used Vision Transformer, and for classification, they used ResNet-9 deep learning models. These models produced comparable outcomes compared to other pre-trained models (Vallabhajosyula et al., 2024).

Rice plant leaf disease detection was performed using a Deep Spectral Generative Adversarial Neural Network (DSGAN^2^). By introducing GAN, the researchers increased the size of the image dataset, which in turn improved the model’s performance in plant disease detection. However, the approach needs to be tested further on other crops to analyze whether the method is scalable or not (Cheemaladinne and Reddy, 2024). A.K. Singh et al. developed LeafyGAN, a deep learning model that is a combination of Pix2PixGAN for segmentation and CycleGAN for image translation. With the implementation of these two methods, the researchers successfully generated synthetic images to balance the image dataset. These images were then fed into the lightweight MobileViT and trained for image classification on two different datasets, PlantVillage and PlantDoc. This model performed well on the PlantVillage dataset but not on the PlantDoc dataset, where the model achieved only 75% accuracy (Longo et al., 2024). An integrated VARMAx–CNN–GAN was proposed by researchers for tomato leaf disease detection and management. It is a deep learning model integrating CNNs, GANs, and Vector AutoRegressive Moving Average processes with eXogenous regressors (VARMAx). CNN was used for feature extraction, GAN for generating synthetic images, and the VARMAx component for improved disease classification (Mahim et al., 2024).

Amreen Abbas et al. combined Conditional Generative Adversarial Networks (C-GANs) and a pre-trained DenseNet121 model. A Conditional GAN was used to generate the images, and DenseNet121 for disease classification. Their study aimed to increase the size of the limited image dataset using data augmentation, which in turn helped their model classify diseased tomato leaves in multiclass datasets. However, the model used for classification was a pre-trained one (Sultana et al., 2024). To address the class imbalance problem in graph-structured data for node classification tasks, Bojia Liu et al. introduced Class Distribution-aware Conditional Generative Adversarial Network (CDCGAN). This model aimed to generate diverse and distinguishable minority nodes based on a C-GAN-based minority augmentation module and a class distribution awareness module that extracts node embeddings. The model allows greater generalization ability for different GNN encoders during testing, but it may not be applicable in dynamic graph scenarios (Alhammad et al., 2025).

XAI is a subset of AI that emphasizes growth and importance across various domains. XAI highlights important features and provides a multidisciplinary approach for researchers. Ethical, human-centered, and holistic approaches should be used in developing XAI systems (Nigar et al., 2024). A hybrid model was proposed using Vision Transformer and GRU for Alzheimer’s disease detection and classification. In that study, they incorporated XAI methods to enhance the model’s interpretability in decision-making. LIME, SHAP, and Attention Map XAI techniques were used to provide a transparent view of the AI’s reasoning (Chettri et al., 2024). XAI-FruitNet was a fruit classification model integrated with average and max-pooling techniques. This improved feature discrimination and incorporated Explainable AI to enhance model transparency through Grad-CAM. Grad-CAM explains the most contributing parts of an image, which helps in making classification decisions (Liu et al., 2021).

Transfer learning was used in the pre-trained Xception model to classify and predict potato leaf diseases. These results were interpreted using one of the Explainable AI techniques, Grad-CAM, which extensively emphasizes the core area of the leaf through visualization, thereby addressing the critical gap in existing research (Jannat et al., 2025). Natasha Nigar et al. compared four deep learning models—CNN, MobileNetV2, EfficientNetB0, and ResNet-50—and found that EfficientNetB0 outperformed the other three in predicting plant leaf diseases. The XAI-based LIME technique was included in their study for the interpretation of the proposed model. LIME was employed to provide a visual explanation of the predictions made by the model (Dogra et al., 2023). The Vision Transformer model was proposed for the PlantVillage dataset, and this work has achieved close to 98% accuracy with data augmentation by balancing the different classes of data (Lu et al., 2023). Another Transformer-based work was proposed based on multi-scale feature fusion, which had a better generalization outcome compared to the other state-of-the-art CNN-based models (Mahadevan et al., 2024). An ensemble-based customized EfficientNet model was proposed for disease detection in plants like corn, potato, and tomato. This model has achieved close to 99% accuracy with the least misclassification rate (Singh et al., 2024).

Haridasan, A. et al. used CNN and SVM to detect five different rice crop leaf diseases of the paddy dataset and attained 91% accuracy (Haridasan et al., 2023). Deng, R. et al. proposed an ensemble model with DenseNet-121, SE-ResNet-50, and ResNeSt-50 to predict the paddy dataset with six diseased classes, namely, rice leaf blast, false smut, neck blast, sheath blight, bacterial stripe disease, and brown spot, achieving 91% accuracy (Deng et al., 2021). Elmitwally, N. S. et al. chose bacterial leaf blight, brown spot, and leaf smut leaf disease classes and trained using AlexNet for prediction with 99% accuracy, but only three classes were chosen (Elmitwally et al., 2022). Upadhyay, S. K. and Kumar, A. used the Kaggle rice leaf disease dataset with three diseased leaf classes and one healthy class and predicted the leaf disease using a deep learning-based CNN model with 99.7% accuracy, but the number of classes chosen was only four (Upadhyay and Kumar, 2022). Gaurav Shrivastava and Harish Patidar proposed SVM with ANN for predicting three classes of the Kaggle rice dataset with 91% accuracy (Shrivastava and Patidar, 2022). Rajpoot, V. et al. proposed a VGG-16-based transfer learning Faster R-CNN model for predicting bacterial leaf blight, brown spot, and leaf smut diseased leaf datasets with 97.3% accuracy; however, only three classes of diseased leaves were chosen (Rajpoot et al., 2023). Bhakta, I. et al. used a bacterial leaf blight rice dataset, which is a binary classification, with CNN and obtained 95% accuracy (Bhakta et al., 2023). T. Daniya and S. Vigneshwari proposed a Rider Henry Gas Solubility Optimization (RHGSO)-based deep neuro-fuzzy network (DNFN) model for predicting three classes—BLB, blast, and brown spot disease leaf datasets—and attained 93% accuracy (Daniya and Vigneshwari, 2023).

The Paddy Doctor dataset was used in many of the studies employing various deep learning-based models. Villegas-Cubas et al. deployed the InceptionV3 model to classify and predict nine classes of diseased leaf images and one healthy class, achieving an accuracy of 88% (Villegas-Cubas et al., [[NoYear]]). Quan T. H. and Hoa N. T. proposed the RiceNet Classification model for classifying 10,407 images and attained 93.8% accuracy (Quan and Hoa, 2024). For the same dataset, Tasnim F. et al. proposed hybrid association rule mining (ARM) with logistic regression and achieved 92.8% accuracy (Tasnim et al., 2025). Garg et al. implemented the EfficientNet model for the Paddy Doctor dataset with an accuracy of 91% (Garg et al., 2023). Klair et al. selected several classes from the same dataset and implemented different models like ConvNet, ResNet, and EfficientNet, achieving accuracies of 87%, 91%, and 94%, respectively (Klair et al., 2024). From the Plant Doctor dataset, only one diseased class (white stem borer) and one healthy leaf class were chosen, and a ViT model was proposed to predict plant leaf diseases for binary classification with 96% accuracy (Felicita and Kavitha, 2024). **Table 1** summarizes the literature review.

**Table 1:** Summary of literature survey.

Author name and year	Dataset	Algorithm used	Accuracy in %
(Maurício et al., 2023)	Brown spot	CNN-VGG16	93%
(Longo et al., 2024)	PlantDoc dataset	LeafGAN	75.72%
(Haridasan et al., 2023)	Paddy dataset of 5 diseases	CNN and SVM	91%
(Deng et al., 2021)	Paddy dataset with rice leaf blast, false smut, neck blast, sheath blight, bacterial stripe disease, and brown spot diseased classes	Ensemble model DenseNet-121, SE-ResNet-50, and ResNeSt-50	91%
(Elmitwally et al., 2022)	Bacterial leaf blight, brown spot, and leaf smut leaf disease classes	AlexNet	99%
(Upadhyay and Kumar, 2022)	Kaggle rice leaf disease dataset with 3 diseased leaf classes and 1 healthy class	CNN	99.7%
(Shrivastava and Patidar, 2022)	Kaggle rice dataset classified for 3 classes	SVM with ANN	91%
(Rajpoot et al., 2023)	PlantVillage dataset—4 classes of maize diseased leaves	Multi-Model Fusion Network (MMF-Net), AlexNet, VGG-16, and ResNeXt	99%
(Bhakta et al., 2023)	Bacterial leaf blight, brown spot, and leaf smut diseased leaf dataset	VGG-16-based transfer learning for Faster R-CNN	97.3%
(Daniya and Vigneshwari, 2023)	Bacterial leaf blight rice dataset—binary class	CNN	95%
(Villegas-Cubas et al., [[NoYear]])	PlantVillage dataset	Hierarchical Convolutional Neural Network (H-CNN)	93%
(Quan and Hoa, 2024)	PlantVillage dataset	ResNet18	94.4%.
(Tasnim et al., 2025)	27 different species of plant leaf dataset of PlantDoc dataset without rice plant	EfficientNet	80.19%
(Garg et al., 2023)	Bacterial leaf blight (BLB), blast, and brown spot diseases leaves dataset	Rider Henry Gas Solubility Optimization (RHGSO)-based deep neuro-fuzzy network (DNFN)	93%

Based on the referred literature, we have formulated our research problem and the model to address the identified gaps, and the next section presents the detailed methodology along with the proposed model. Some of the key findings from the review of literature are that many previous works have considered a smaller number of diseases and achieved better accuracy. Many studies have focused on BLB, blast, and brown spot. Therefore, we have arrived at our problem statement involving six prominent diseases of the rice crop and aim to propose a model with better accuracy that can be compared with other classifiers proposed with fewer classes. Furthermore, the superiority of the ViT-based classifier, which has been demonstrated in computer vision problems, will be explored in our model. The proposed model and the methodologies used are discussed in the next chapter.

## 3 Methodology used

**Figure 1:**
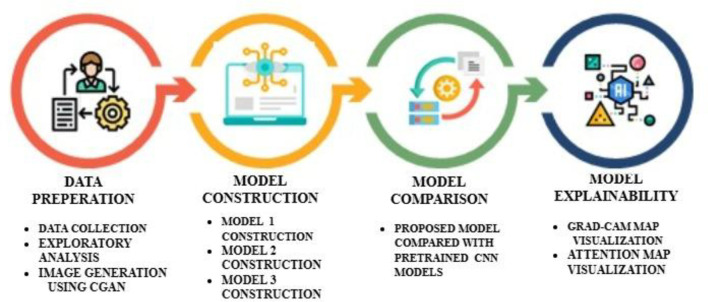
Overall system flow diagram of the proposed models.

This section provides details of the data used, the methodology employed, and the result analysis. **Figure 1** depicts the step-by-step process involved in developing the proposed work. Step 1, data preparation, starts with data collection and data pre-processing. The step continues with generating synthetic images using a C-GAN, followed by normalization and augmentation. The second step involves model construction and implementation of the Vision Transformer model with a self-attention mechanism and multi-layer perceptron (MLP) classifier. For the training and testing of the model, the dataset is split in an 80:20 ratio. The proposed models are implemented with variations in the configuration of hyperparameters to analyze and evaluate their impact on model performance. The third step is to compare the performance of the proposed models with pre-trained CNN models. Finally, the performance of the proposed model is interpreted with the help of techniques like Grad-CAM and Attention Map Visualization to evaluate the trustworthiness of the model.

### 3.1 Dataset description

**Figure 2:**
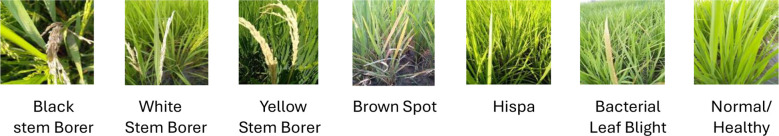
Sample images of healthy and diseased rice plant leaves.

In this work, we considered paddy leaf images from the Paddy Doctor dataset, which is available on IEEE DataPort. This dataset incorporates 12 classes of diseased paddy leaves and a class of healthy paddy leaves. There are a total of 16,225 images after cleaning and manual annotation from over 30,000 images collected from the surroundings of Tirunelveli district, Tamil Nadu, India (Petchiammal et al., 2024). Among these 12 classes, we have considered six diseases that have a major impact on overall crop yield production. These six classes are black stem borer, white stem borer, yellow stem borer, brown spot, hispa, and BLB, along with the class of healthy leaves to enable efficient classification. The number of images chosen for this work before balancing the dataset for each class is tabulated in **Table 2**. The sample test images of all six diseased classes and the healthy class are depicted in **Figure 2**.

**Table 2:** Dataset description.

Class	Number of images
Black stem borer	506
White stem borer	1,250
Yellow stem borer	765
Brown spot	1,257
Hispa	2,151
Bacterial leaf blight (BLB)	648
Normal/healthy	1,250

### 3.2 Proposed architecture

A hybrid Conditional GAN with Vision Transformer is implemented in this work for improved plant disease classification of a multiclass rice crop dataset. For data preparation, we use seven classes of paddy leaf images, which are analyzed through exploratory data analysis. From the exploratory data analysis, we found that the performance of the Vision Transformer model is comparatively low when the dataset is imbalanced. To balance this dataset, the Conditional GAN algorithm is used to increase the size of the classes. The Conditional GAN model is customized with a U-Net generator and PatchGAN discriminator to generate synthetic images. These are then given as input to the Vision Transformer multiclass classification model for the augmentation of the entire boosted dataset. These augmented images are split into patches, flattened into 1D linear projections, and appended with positional embeddings.

Once augmentation and patch embedding are completed in the pre-processing stage, they are given as input to the transformer encoder. The transformer encoder comprises layer normalization for stabilizing the training process, multi-head attention layers to capture and layer the dependencies of different patches of an image, and an MLP to further process the image by introducing non-linear functions. The outputs of the MHA and MLP are added through skip connections and fed back to the previous layer output. Finally, the output of the transformer encoder is fed into the classification head to classify the different classes of diseased plant leaves and healthy leaves.

In this work, two different configurations of the model are implemented to enhance the model’s performance. The first, RG-ViT, model is implemented with eight layers of transformer encoders with ReLU activation functions. Activation functions are used in deep learning models to learn complex patterns through their non-linear functions. In the RG-ViT model, we use only ReLU activation functions at both the encoder layers and the final classifier layer. To further enhance the model’s performance, the second model, GRG-ViT, is configured with 12 layers of transformer encoders. The second variation from model 1 is the type of activation function used. In this model, both Gaussian Error Linear Unit (GELU) and ReLU activation functions are included: the ReLU activation function is used within each transformer encoder, and the GELU activation function is used in the classification head, as it captures global dependencies better than ReLU.

**Figure 3:**
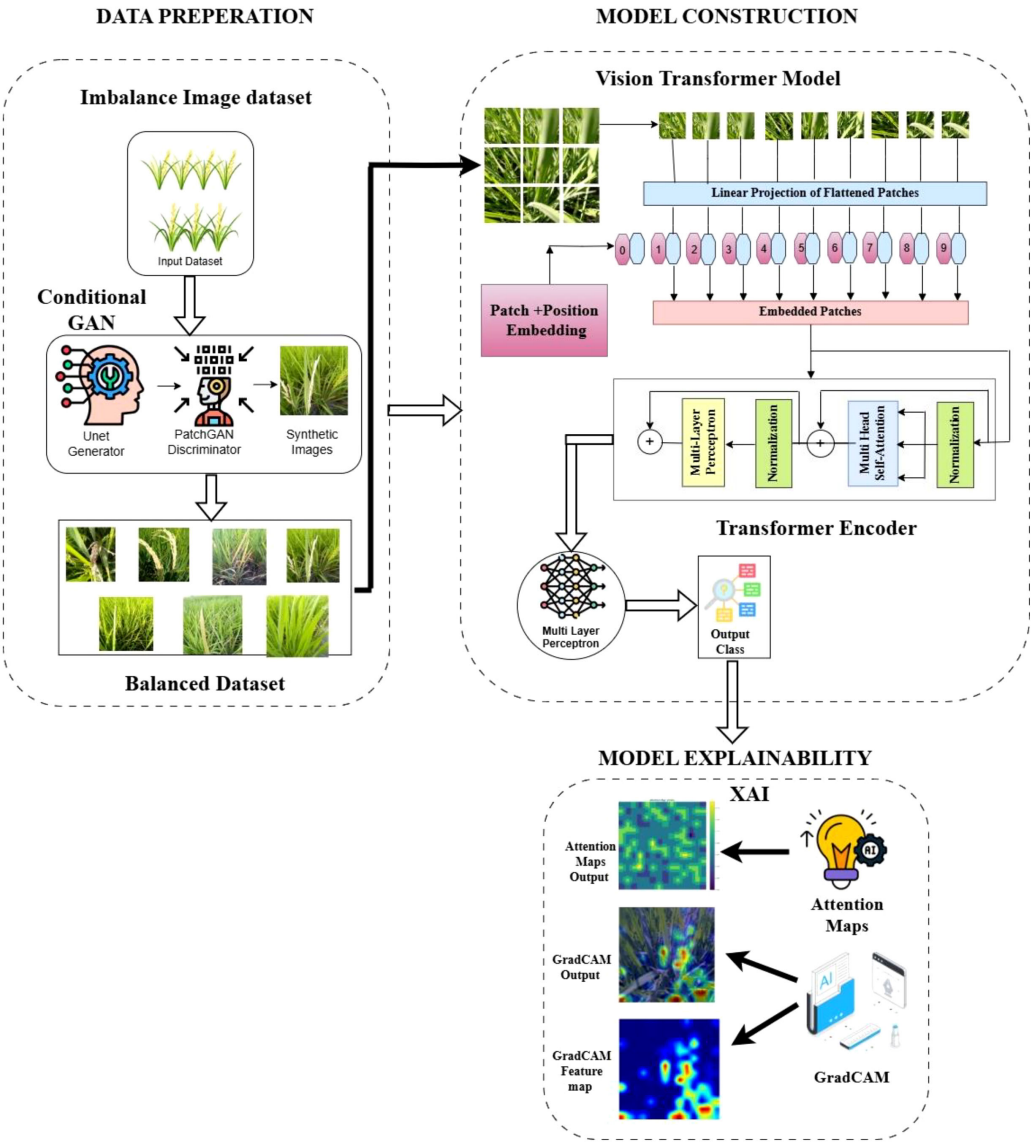
The proposed architectural diagram for multiclass classification.

The outputs of the Vision Transformer model are illustrated using state-of-the-art XAI techniques like Grad-CAM and Attention Mapping. These visualization methods capture the core areas where the Vision Transformer focuses on making classification decisions. Attention Mapping visualization is used to visualize and extract the multi-head attention patterns. Grad-CAM mapping highlights specific regions that influence class prediction by generating class activation maps. These two techniques are included in this work for both qualitative and quantitative analyses, particularly for understanding model prediction capability, identifying biases, and enhancing model design and performance. The architecture of the proposed GRG-ViT model is depicted in **Figure 3**.

### 3.3 Conditional GAN architecture for data balancing

In this proposed model, a C-GAN, a GenAI method, is implemented to generate synthetic images similar to the original images. The dataset used here is imbalanced, with one class having 2,100 images, whereas another class has only 506 images. To improve the performance of the proposed Vision Transformer model, the imbalanced classes need to be balanced. Conditional GAN is an extended version of a GAN with conditioning applied to both adversarial models. The condition is the auxiliary information (y), which influences both the generator (G) and discriminator (D) by including it. This can be expressed as an objective function for the real image x and the condition y (here, y is the class label) over a noise vector z, determined using a min–max function as shown in Equation 1 (Goodfellow et al., 2014).

(1) $$\underset{G}{\min}\underset{D}{\max}C_{GAN}\left(G,D\right)=\;E_{x,y}\left[logD\left(x,y\right)\right]+E_{y,z}\left[\log\left(1-D\left(G\left(y,z\right),y\right)\right)\right]$$

**Figure 4:**
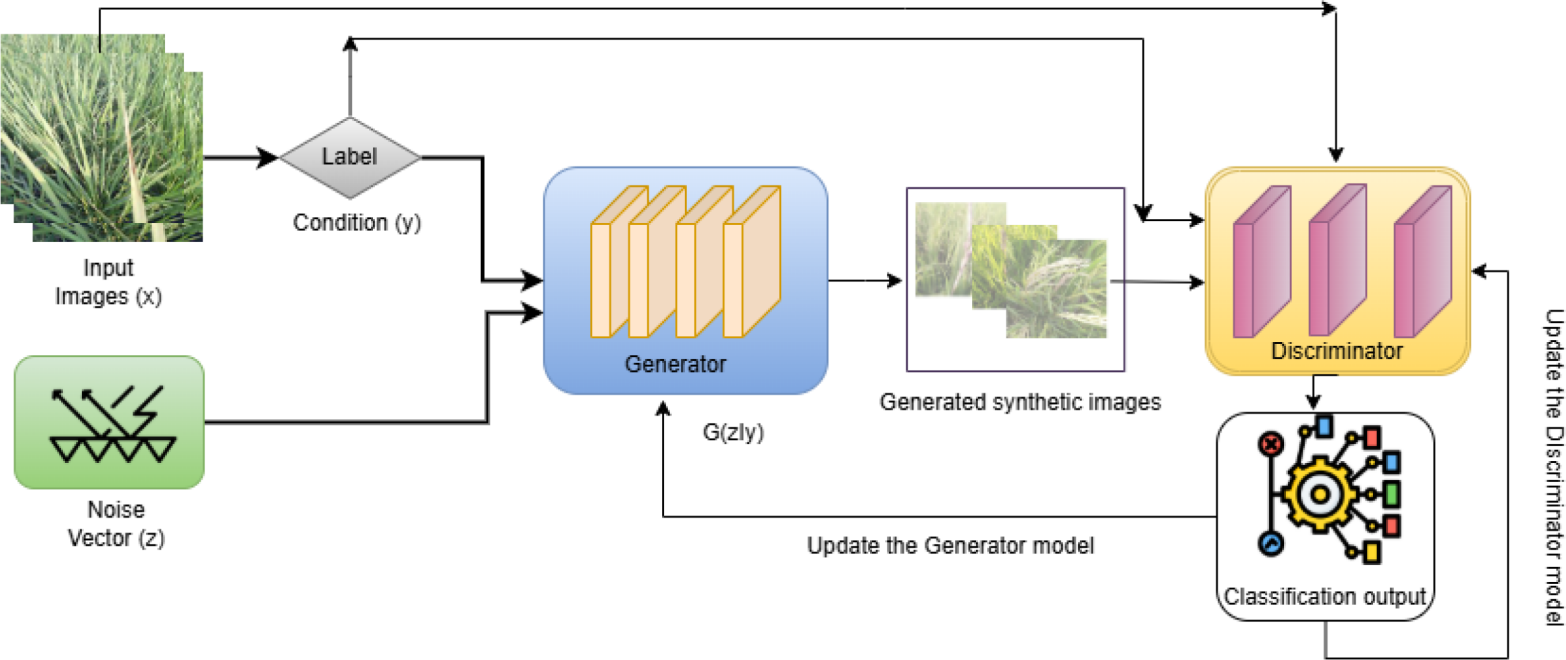
Conditional GAN architecture with U-Net generator and Patch-GAN discriminator.

**Figure 4** depicts the architectural representation of the Conditional GAN, with the input images x concatenated with the label y and noise vector z fed as input to the U-Net Generator to generate synthetic images. These generated images, along with the input image and condition, are given as input to the PatchGAN discriminator to classify the input and synthetic images. The classified output is then used to update both the discriminator and generator to train the Conditional GAN model and improve its performance.

#### 3.3.1 Generator and discriminator

The generator used in the Conditional GAN for the proposed model is based on the U-Net architecture, which exclusively performs the image generation task while preserving spatial information. The U-Net generator generates images with the implementation of a contracting path (encoder) and an expansive path (decoder). The output of both the encoder and decoder is concatenated using skip connections to generate the final image. The encoder down-samples the input images by implementing seven blocks of convolutional layers, batch normalization, and LeakyReLU activation functions. By doing this, it reduces the spatial dimensions of the input image, lowering the resolution from 256 × 256 down to 2 × 2, while the number of channel features increases. For every corresponding encoder layer, the decoders progressively up-sample the encoded features from 2 × 2 to 256 × 256. These are then concatenated with the down-sampled feature maps of the encoder using skip connections at every block level. The final block of the decoder implements a tanh activation function to generate output values normalized between [−1, +1] for *l* layers of encoder and decoder. Output *G*(*y*) of the U-Net generator is represented as in Equation 2.

(2) $$G\left(y\right)=tanh\left(W_{out}*f\left(W_{d}^{\left(l\right)}*Concat\left(upsample\left(u_{d}^{\left(l+1\right)}\right),f\left(W_{e}^{\left(l\right)}*u_{e}^{\left(l-1\right)}+b_{e}^{\left(l\right)}\right)\right)+b_{d}^{\left(l\right)}\right)+b_{out}\right)$$

where *u_e_* is the generator encoder, *u_d_* is the generator decoder, *f* is the LeakyReLU activation function, *W* is the weight, and *b* is the bias.

The PatchGAN discriminator is used, as it classifies patches of an image rather than the entire image. This method captures high-frequency details and produces the final output by averaging all patch responses. The discriminator D obtains two inputs: either the input image (x) or the image generated by the U-Net generator G(y, z), concatenated with the condition (label y). Four blocks of convolutional layers, batch normalization, and LeakyReLU activation functions are used to process the concatenated input for down-sampling. The final block uses the sigmoid activation function to produce the output in a 2D matrix form, where each element represents a patch. The convolutional output from down-sampling the output of the U-Net generator for *l* layers in the discriminator is represented as in Equation 3.

(3) $$D\left(x,y\right)=\sigma \left(W_{D}^{final}*u_{D}^{\left(l\right)}+b_{D}^{final}\right)$$

where * denotes the convolution operations, $u_{D}^{\left(l\right)}$ is output feature map of discriminator at layer *l*, *W* is filter weight associated with the final layer, f is LeakyReLU activation function, and σ is for sigmoid activation function.

As mentioned in Equation 1, the Conditional GAN generator generates synthetic images, which are evaluated by the discriminator. The loss function of the discriminator in a Conditional GAN should be maximized to categorize real and synthetic images. The loss function of the generator should be minimized so that it can generate images matching the input image x. After calculating the losses from both networks, gradients are computed to update the model parameters. Once training is completed with the Conditional GAN, synthetic images are generated for the minority classes and added to the training set to convert the imbalanced dataset into a balanced one. In this work, the synthetic images are added to the bacterial leaf blight, black stem borer, and yellow stem borer classes.

### 3.4 Vision Transformer model for plant leaf detection

Vision Transformer is the latest development among deep learning models, specifically designed for computer vision tasks. It uses transformers as its backbone architecture, which has a unique capability called the self-attention mechanism. This model can identify the dependencies and relationships between image patches, irrespective of their distance. The detailed process of the Vision Transformer is illustrated in the architectural diagram in **Figure 3**. The seven classes of plant disease images are converted into patches, embedded into patch embeddings, and then flattened using linear projections. Patch embeddings, added with positional embeddings, are given as input through a stacked transformer encoder, which produces a refined set of classification (CLS) tokens. The CLS token output represents the summary of the entire image, which is used for detection, segmentation, or classification. The encoder output is given to an MLP, where it enhances the representation of CLS tokens by introducing non-linear transformations to extract more expressive features. Finally, the classifier of the Vision Transformer predicts the different types of diseased leaves and healthy leaves in the multiclass dataset.

#### 3.4.1 Patch extraction and positional embedding

The images collected are resized to 72 × 72, then pre-processed, and flattened into 2D patches, as the transformer model can only receive input as a sequence of one-dimensional tokens. For instance, in this work, the resolution of the input image x is taken as 72 × 72 with three channels and a pixel P of size 6 × 6. Therefore, we will obtain N = (72 × 72)/(6 × 6) = 144 image patches after flattening. Positional encoding is added to preserve the original spatial information. These patches are then mapped to a lower-dimensional trainable linear projection to generate patch embeddings. Patch embeddings are then added with positional information to retain the original position of each image, producing positional embeddings. These positional embeddings are given as input to the transformer encoder, with classification tokens added to the patch embeddings for the final representation of the different classes. RGB image *x* with resolution of (H, W) over C number of channels is represented, as $x\;\in R^{H\times W\times C}\;$ is converted into N non-overlapping image with (P, P) resolution of patches $x_{p}\in R^{N\times \left(P^{2}•C\right)}$, where *N* = (*HW*/*P*^2^). These patches are mapped to linear projection D to obtain $E\;\in R^{N\times \left(P^{2}•C\right)\times D}$ patch embedding *p_i_*. Once these patch embeddings are known, the position embeddings *E_pos_* are added to know their spatial information $E_{pos}\epsilon \in R^{\left(N+1\right)\times D}$. The sequence embedding vector t in Equation 4 is obtained once the position embeddings are calculated after prepending the classification token *x_class_*.

(4) $$t=\left[x_{class};\;x_{1}E;\;x_{2}E;\ldots \ldots \ldots x_{N}E\right]+E_{pos}$$

This *t* has been given as input to the transformer encoder.

#### 3.4.2 Transformer encoder and attention mechanism

The transformer encoder in the ViT architecture consists of L layers, with every layer having an alternating multi-head self-attention module (MSA) and feed-forward MLP module. Every layer in the transformer encoder has a normalization layer to give the normalized inputs to the other two modules through the residual connection. The input given to the MSA module of every *i* layer of transformer is *t^i^*, which is given as input to the multi-layer perceptron to obtain.

(5) $$t^{i+1}=MLP\left(MSA\left(LN\left(t^{i}\right)\right)\right)+t^{i}$$

Multiple self-attention mechanisms are employed within each multi-head self-attention module. The self-attention mechanism has the capability to enable the model to learn and understand the relationships and dependencies in every patch of an image. This is performed by assigning scores based on the importance of the most relevant information. The self-attention mechanism includes three key parameters—Query (Q), Key (K), and Value (V)—applied to every individual patch of an image. The Query of one patch pays attention to all other patches of an image to analyze which patch is more relevant and important with respect to its representation. The Key helps to determine how each patch matches the respective Query, and the Value is used to calculate the actual information or features of the patches (Dosovitskiy et al., 2020). The single model dimension for each head can be linearly projected for h times in different projections of query, key, and value as *d_q_*, *d_k_*, and *d_v_*, respectively, to compute multi-head self-attention as in Equation 6 For different projection values, the corresponding projection metrics for query, key value, and output parameter can be formulated as $W_{m}^{Q}\in R^{d_{model}\times d_{k}}$, $\;\;W_{m}^{K}\in R^{d_{model}\times d_{k}}$, $\;W_{m}^{V}\in R^{d_{model}\times d_{v}}$ and $W_{m}^{O}\in R^{hd_{model}\times d_{k}}$Therefore,

(6) $$MultiHead\left(Q,K,V\right)=Concat\left(head_{1},\ldots ,head_{m}\right)W^{O}$$

#### 3.4.3 where $head_{m}=Attention(QW_{m}^{Q},KW_{m}^{K},VW_{m}^{V}\;$) and $Attention\left(Q,K,V\right)=softmax\left(\frac{QK^{T}}{\sqrt{d_{k}}}\right)V$Multi-layer perceptron and classification

To process the output of the self-attention mechanism and to complement the MSA layer, an MLP is embedded within the transformer encoder. This is given as input to the final classifier, which is a multi-layer perceptron. It captures critical patterns of an image and enhances the representation by transforming the vectors into higher-level dimensions. This transformation is performed by introducing non-linearity to learn complex relationships, implemented using the non-linear activation function GELU.

The MLP output *t^i^* + 1 of Equation 5 is given as the input to the classifier, which is layer normalized to generate the final prediction vector *v.* Furthermore, the SoftMax layer produces class probabilities for all the different rice leaf categories of the classifier.

(7) $$v=LN\left(x_{class}^{i}\right)$$

The output obtained from Equation 7 presents the performance of the ViT classifier over plant disease detection with improved accuracy compared to other conventional deep learning models.

### 3.5 Explainable AI techniques

XAI is a part of AI that helps in understanding the results obtained from different models. AI-based models are often complex and increasingly difficult to interpret, especially when they make decisions at crucial times, whereas AI models can make life-altering decisions (Ashish et al., 2017). XAI is used in this work to gain deeper insight into how the proposed model makes decisions based on the attention mechanism, important features, and activation functions of classes. XAI enables humans to analyze and improve AI system performance, as the models become transparent in nature. In this work, two important XAI techniques are used to interpret how the proposed hybrid Vision Transformer model classifies and predicts diseased leaves from healthy leaves in a multiclass rice crop dataset. The two techniques are Grad-CAM and Attention Maps, both implemented with customized architectures. A specific adaptation is made to the Vision Transformer model to extract gradient-based explainability features and attention mechanisms for visualization.

#### 3.5.1 Attention Maps

Attention Maps are used to visualize the core regions of the input image that are focused on for predictions by the Vision Transformer model with the attention mechanism. These maps highlight important areas and provide a better understanding of the model, supporting more accurate decision-making in the classification and prediction of plant diseases. This is particularly crucial in agricultural disease diagnosis, where decisions must be made based on relevant features with precision.

#### 3.5.2 Grad-CAM maps

Grad-CAM was initially developed for CNNs; in this work, we have adapted it for our proposed classifier using ViT by adopting class-specific gradients to the attention-weighted patch embeddings from the final encoder block. The output gradients are aggregated here, which are then reshaped to patch grids and then interpolated to the input image resolution (Chefer et al., 2021). This highlights the most influential regions of the image for the prediction. Thus, the updated Grad-CAM is used in a Vision Transformer to bridge the gap between the model and human interpretability. This technique provides visual explanations and generates a feature map to visualize the internal features.

## 4 Results and discussion

This section describes the evaluation results in detail with the classification performances of the proposed model. Three stages of comparison are performed to analyze the performance of the proposed work. First, it is compared with the performance of the ViT using an imbalanced dataset, then with the basic ViT architecture without any hyper-tuning for the balanced dataset, and last with the chosen pre-trained models of CNN.

### 4.1 Performance evaluation

To assess the effectiveness and efficiency of a classification model in deep learning, performance metrics play a key role in measuring the computational efficiency, robustness, and the model’s ability to perform its intended tasks. By evaluating performance, we can further optimize the model, detect errors, and identify biases to avoid inaccurate predictions (Pacal, 2024). Performance metrics are particularly important in assessing the classification and prediction of paddy plant diseases.

Accuracy: For a classification deep learning model, accuracy is one of the most important metrics to measure the overall performance of the model. It is crucial in estimating the proportion of correct predictions among overall predictions.

Precision: It is the metric used to forecast the proportion of accurate positives among all predicted positives.

Recall: The next metric is to estimate the proportion of true positive predictions to the actual positive predictions of the model.

F1 score: This acts as a balance between precision and recall, as it calculates the harmonic means of both precision and recall.

Based on the abovementioned performance metrics, the proposed model with seven classes of paddy plant diseases is evaluated, and the obtained results are tabulated.

### 4.2 Experimental configuration of proposed architecture

The hyperparameter settings of this proposed model are illustrated through a series of controlled experiments. Initially, it is executed with the usual ViT and GAN configurations and then optimized to maximize the validation accuracy. By analyzing a wide range of values from 0.0001 to 0.01 for the learning rate and 64–512 for the batch size, the best values are identified, and it is the learning rate of 0.001 for a batch size of 256 is chosen. A value of dropout rate of 0.2 and a weight decay value of 0.0001 are used to reduce overfitting. Twelve transformer layers and four attention heads are identified in order to balance the model complexity and training time. The final configuration is summarized and tabulated in **Table 3**. This experiment is executed on Google Colab using an NVIDIA A100 GPU, 8 GB RAM, 256 GB storage, with an average training duration of approximately 70 to 85 minutes for 100 epochs. The resulting proposed model, occupying only 998 KB on disk, which ensures that it is both lightweight and efficient, also performs inference of a single image in under 0.1 second.

**Table 3:** Hyperparameter settings.

Hyperparameter	Value/setting
Input image size	72 × 72
Patch size	6 × 6
Number of layers	12
Number of heads	4
MLP size	2,048, 1,024
Dropout rate	0.2
Learning rate	0.001
Batch size	256
Weight decay	0.0001
Total epochs	100
Optimizer	AdamW

### 4.3 ViT with an imbalanced dataset

**Figure 5:**
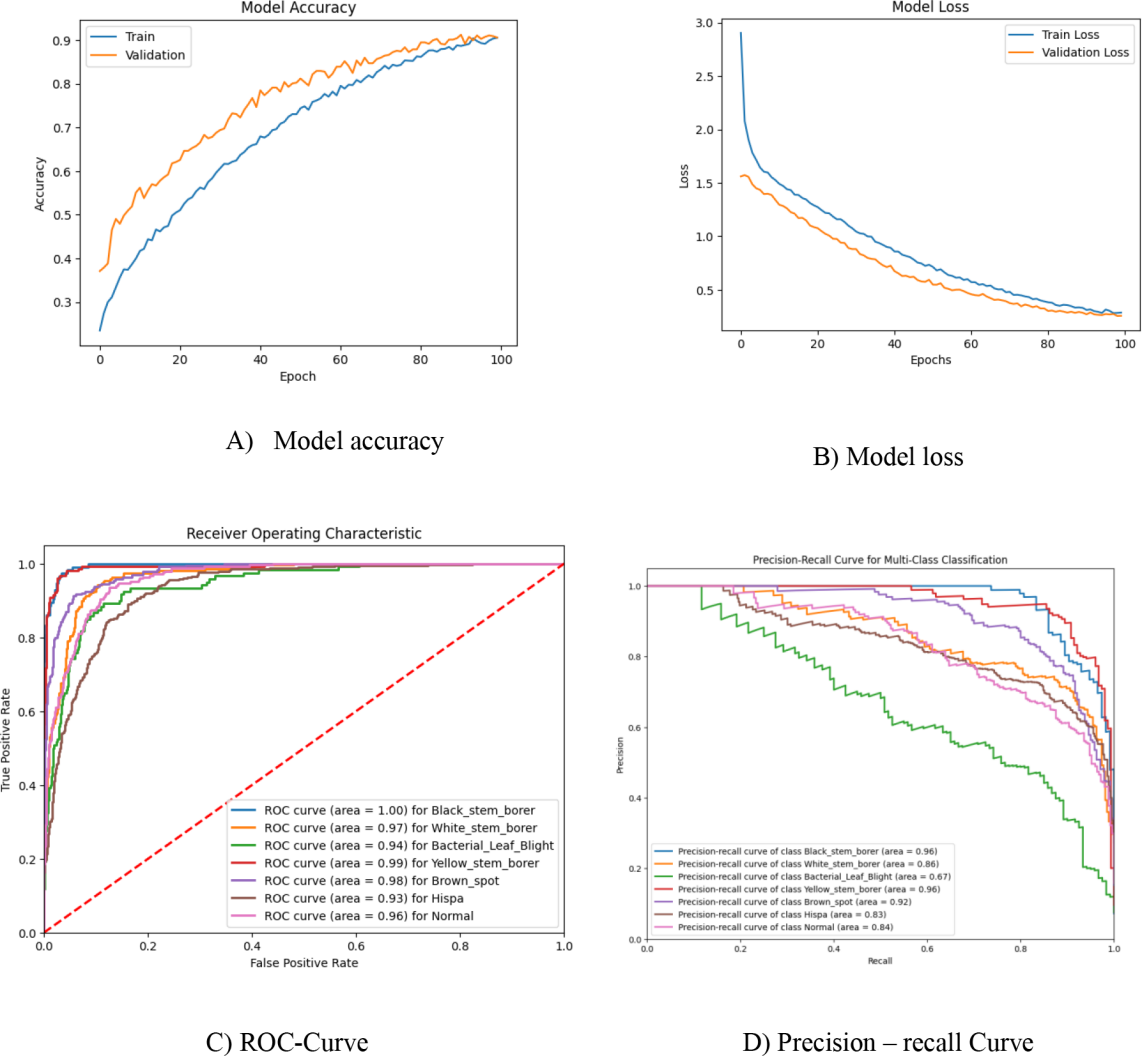
Vision Transformer model for imbalanced dataset.

The Vision Transformer algorithm for multiclass classification in plant disease prediction is applied in this work. The dataset used has 506 black stem borer, 648 bacterial leaf blight, 765 yellow stem borer, 1,250 white stem borer, 1,250 normal, 1,275 brown spot, and 2,150 hispa images. This imbalanced dataset is split into 80% for training and 20% for validation. The same experimental setup is used as in the proposed model and repeated for 100 epochs. As seen in the tabulated results in **Table 4**, there is a significant difference in classification accuracy, precision, and recall, as some classes have images nearing 2,000 and 1,000, whereas others have fewer than 1,000 images. The model accuracy and loss graphs, along with the ROC curve and precision–recall curve for the corresponding models, are shown in **Figure 5**, and the results obtained are listed in **Table 4**.

**Table 4:** Performance evaluation of Vision Transformer classifier for imbalanced dataset.

Classes	Precision	Recall	F1 score	Support	Accuracy
Black_stem_borer	0.96	0.96	0.96	114	91%
White_stem_borer	0.94	0.92	0.93	266	
Bacterial_Leaf_Blight	0.89	0.80	0.84	120	
Yellow_stem_borer	0.92	0.97	0.94	152	
Brown_spot	0.85	0.95	0.90	240	
Hispa	0.90	0.91	0.91	429	
Normal	0.92	0.85	0.88	249	

### 4.4 RG-VIT model (ReLU GAN)

**Figure 6:**
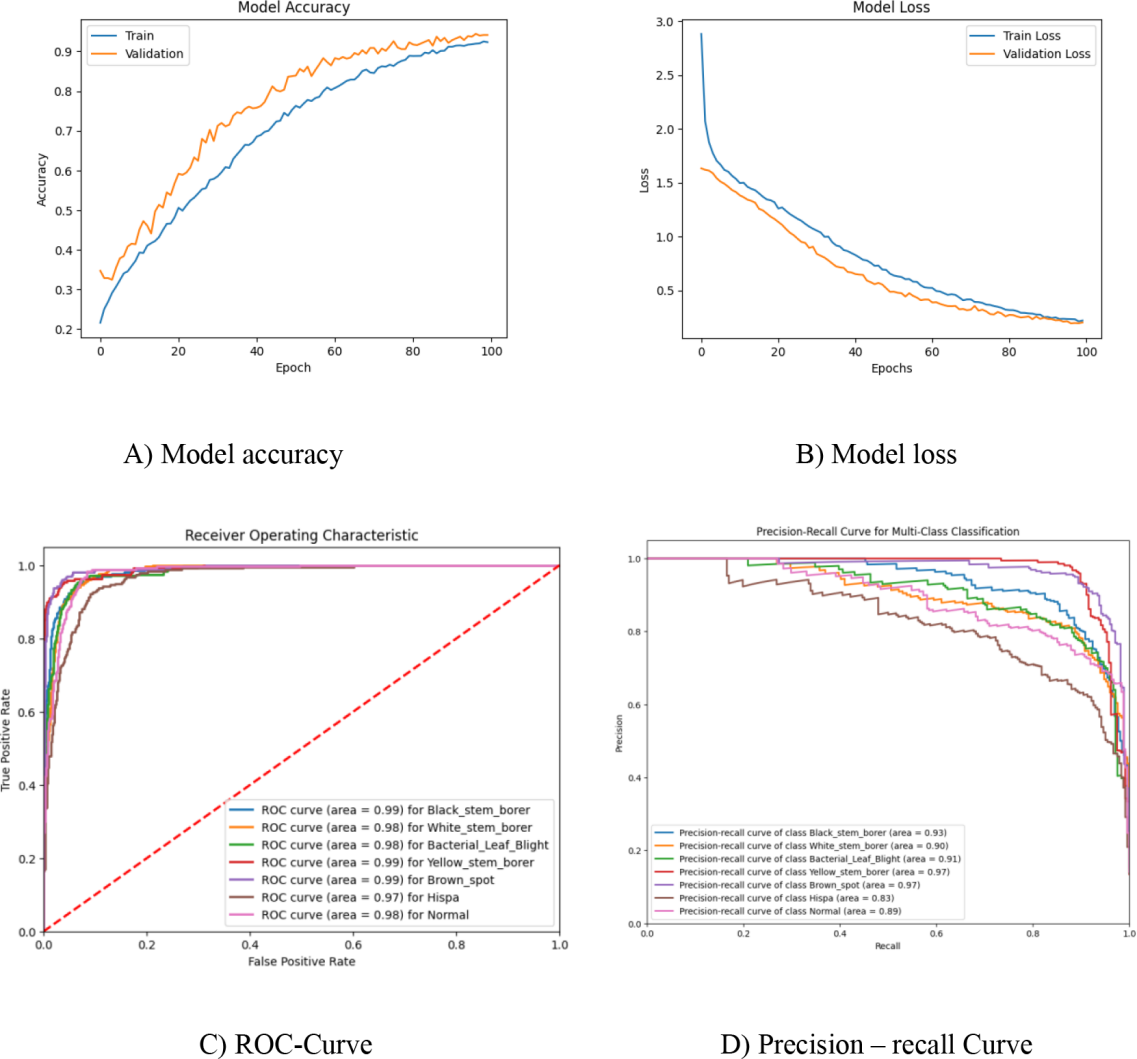
RG-ViT model without hyperparameter tuning.

Model accuracy and precision scores are comparatively low in the previous results, so the imbalanced dataset is balanced using the Conditional GAN deep learning model. Using this, the dataset is balanced, and approximately 1,250 images per class have been obtained through this process. However, the hyperparameters, such as the number of transformer encoder layers and activation functions, are different from those of the other proposed model to analyze classifier performance. Initially, before hyperparameter tuning, the number of transformer encoders used is 8, and the activation function used is ReLU in both the transformer encoders and the multi-layer perceptron classifier. It performs better, with an accuracy of 93% compared to that of the imbalanced dataset. The results are tabulated in **Table 5**, with corresponding model accuracy, loss, ROC, and precision–recall curve graphs depicted in **Figure 6**.

**Table 5:** Performance evaluation of Vision Transformer classifier without hyperparameter tuning.

Classes	Precision	Recall	F1 score	Support	Accuracy
Black_stem_borer	94	95	95	263	93%
White_stem_borer	96	95	95	249	
Bacterial_Leaf_Blight	93	91	92	244	
Yellow_stem_borer	97	97	97	237	
Brown_spot	94	95	94	274	
Hispa	88	93	91	242	
Normal	94	90	92	248	

### 4.5 GRG-ViT model (GELU–ReLU GAN-ViT)

**Figure 7:**
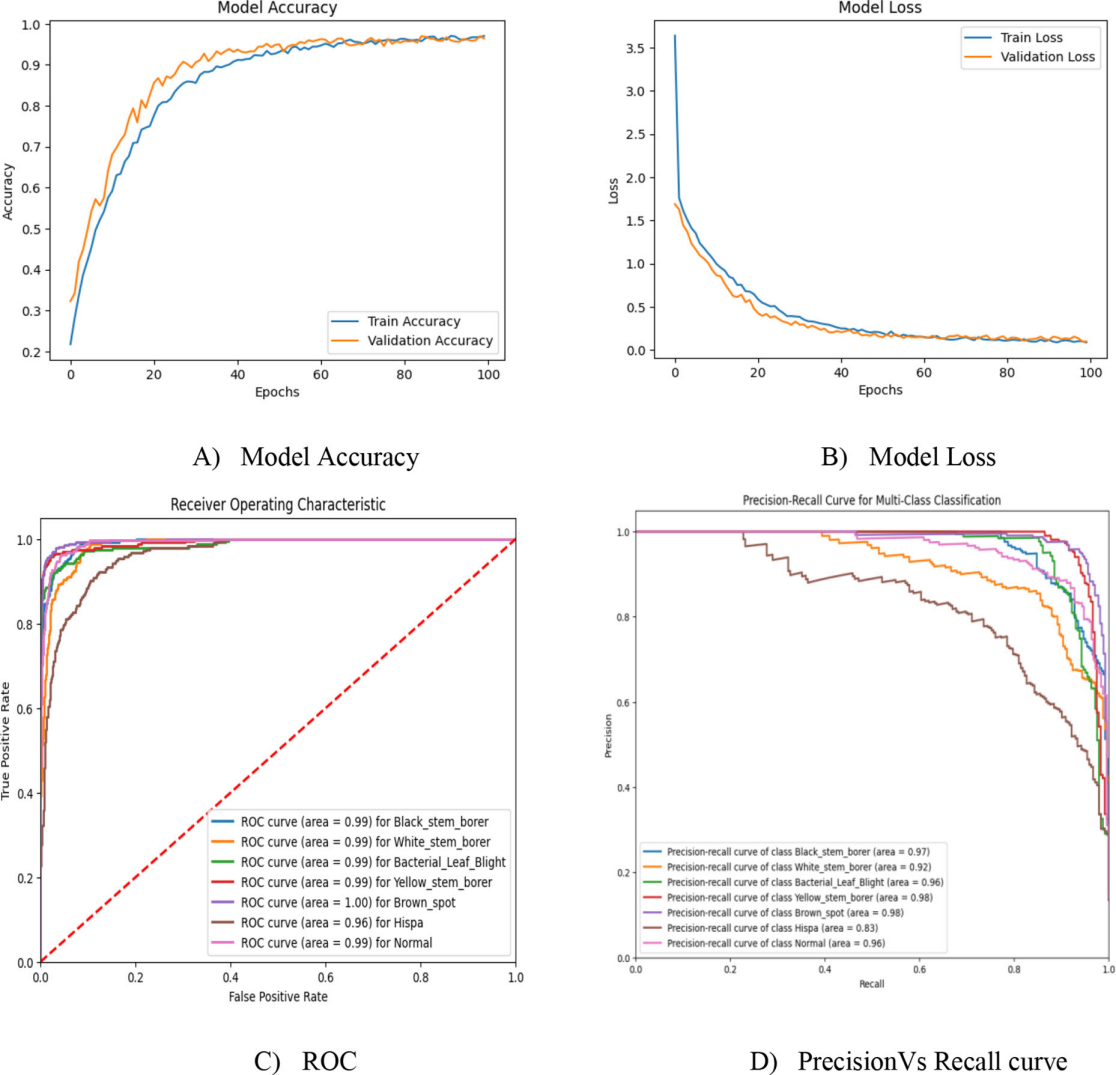
Experimental outcome of the proposed multiclass GRG-ViT model.

**Figure 8:**
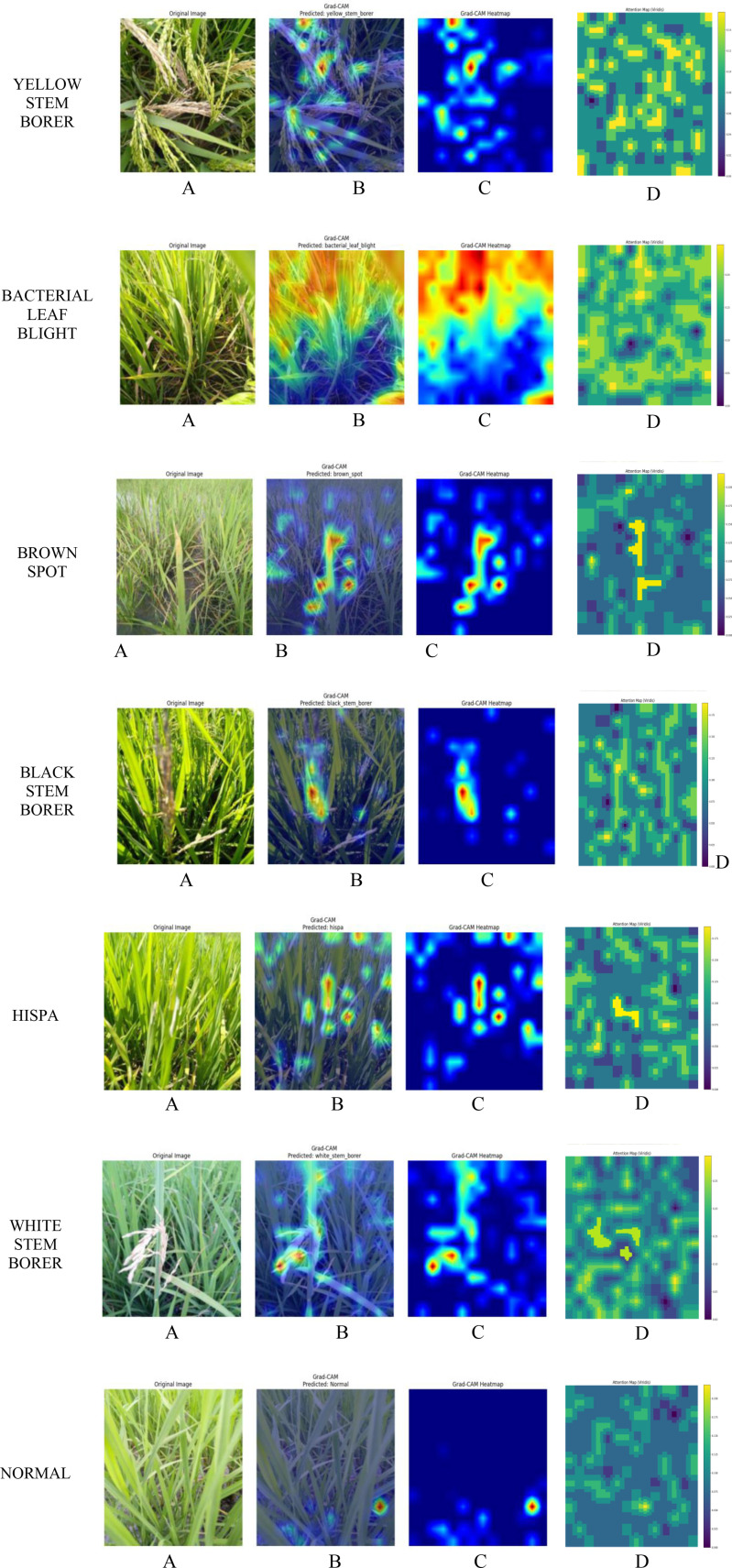
XAI visualization map representing **(A)** original image, **(B)** Grad-CAM map, **(C)** Grad-CAM heatmap, and **(D)** Attention Map.

Usually, ReLU is applied in the intermediate transformer blocks because of its simplicity and lower computational cost, which ensures gradient propagation in deeper layers. GELU used to be chosen in the classification head to provide smoother and non-linear activation. This helps in modeling the complex decision boundaries more effectively. In this work, we have basically performed an ablation study on two different aspects. One is the combined use of ReLU and GELU activation functions, and the other is the effect of varying transformer encoder depth on its classification performance. The hyperparameter tuning includes the number of transformer encoder layers used in the implementation and the activation function. The total transformer encoder layers implemented in this work are 12, with a ReLU activation function in every layer. For the final classifier, the GELU activation function is used to classify the diseased leaves and healthy leaves based on their global dependencies. Combining both ReLU and GELU results in the improvement of both the feature and the decision (Hendrycks and Gimpel, 2016). The per-class performance of the GRG-ViT model is tabulated in **Table 6**, and it is depicted with model accuracy, model loss, ROC curve, and precision *vs*. recall curve in **Figure 7**. Finally, the experimental results are explained with the Attention Map and Grad-CAM map in **Figure 8**.

**Table 6:** Performance evaluation of GRG-ViT model classifier.

Classes	Precision	Recall	F1 score	Support	Accuracy
Black_stem_borer	0.99	0.97	0.98	263	96.74%
White_stem_borer	0.96	0.98	0.97	249	
Bacterial_Leaf_Blight	0.96	0.96	0.96	244	
Yellow_stem_borer	0.98	0.98	0,98	237	
Brown_spot	0.98	0.98	0.98	274	
Hispa	0.95	0.93	0.94	242	
Normal	0.95	0.97	0.96	248	

**Figure 9:**
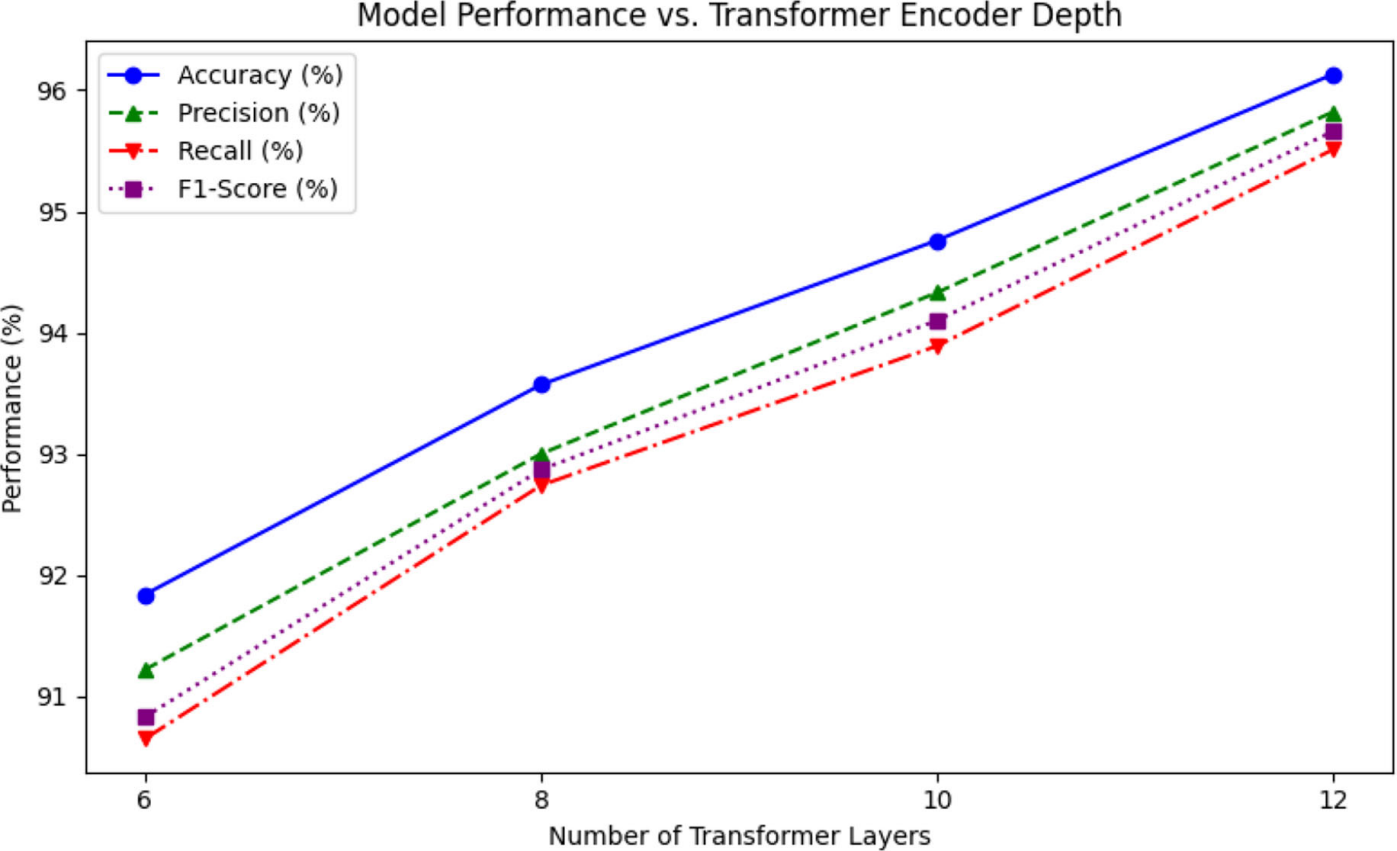
GRG-ViT model’s performance *vs*. transformer depth.

Furthermore, we investigate the impact of the usage of transformer encoder depth on GRG-ViT’s holistic performance. **Table 7** shows the results of different numbers of encoder layers, as well as in **Figure 9**.

**Table 7:** GRG-ViT performance across transformer depths.

No. of transformer layers	Accuracy (%)	Precision (%)	Recall (%)	F1 score (%)
6	91.84	91.22	90.65	90.83
8	93.57	93.00	92.74	92.87
10	94.76	94.33	93.89	94.10
12 (proposed)	96.13	95.82	95.51	95.66

### 4.6 Pre-trained CNN models

**Figure 10:**
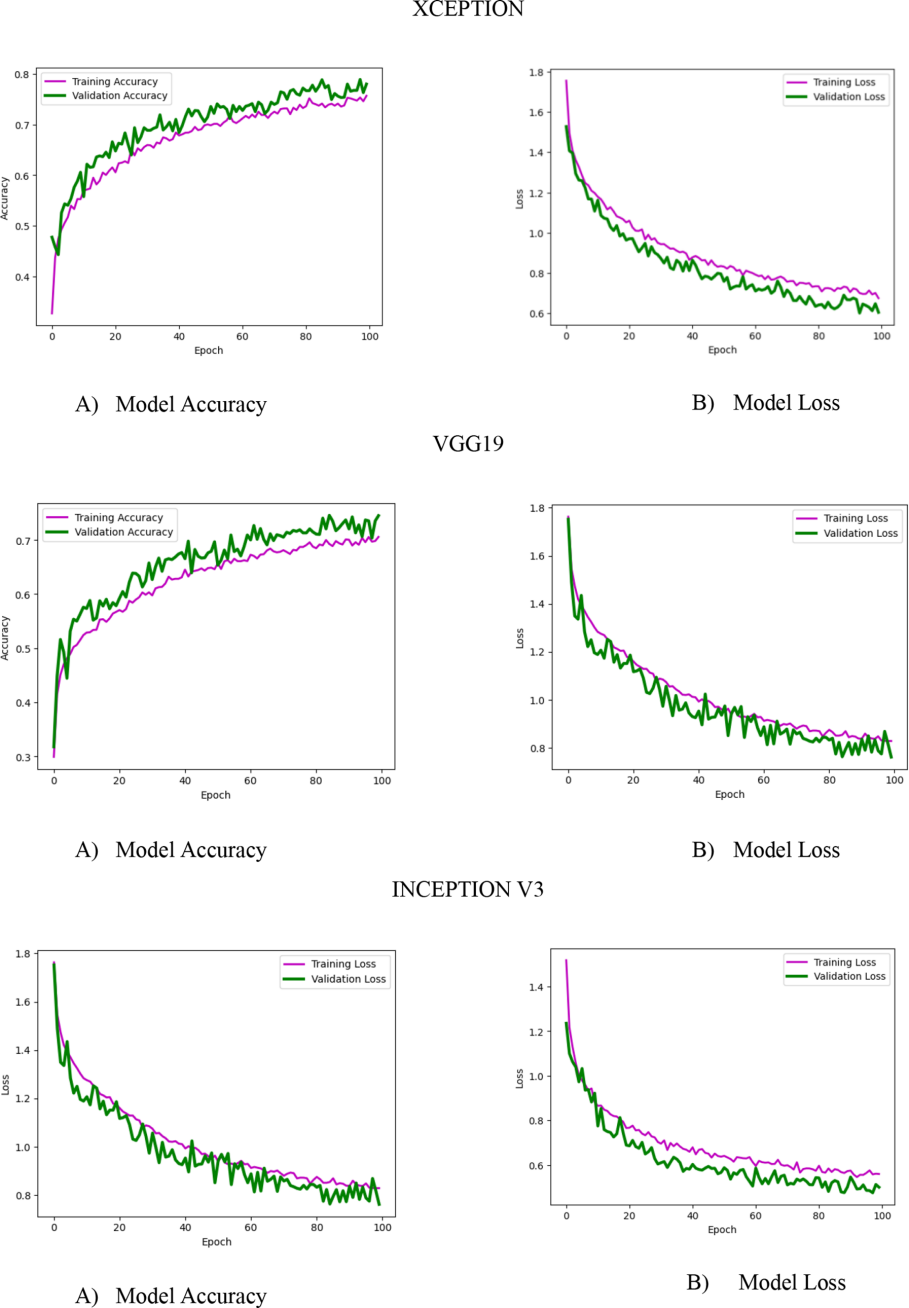
Accuracy and loss of the pre-trained CNN model.

**Figure 11:**
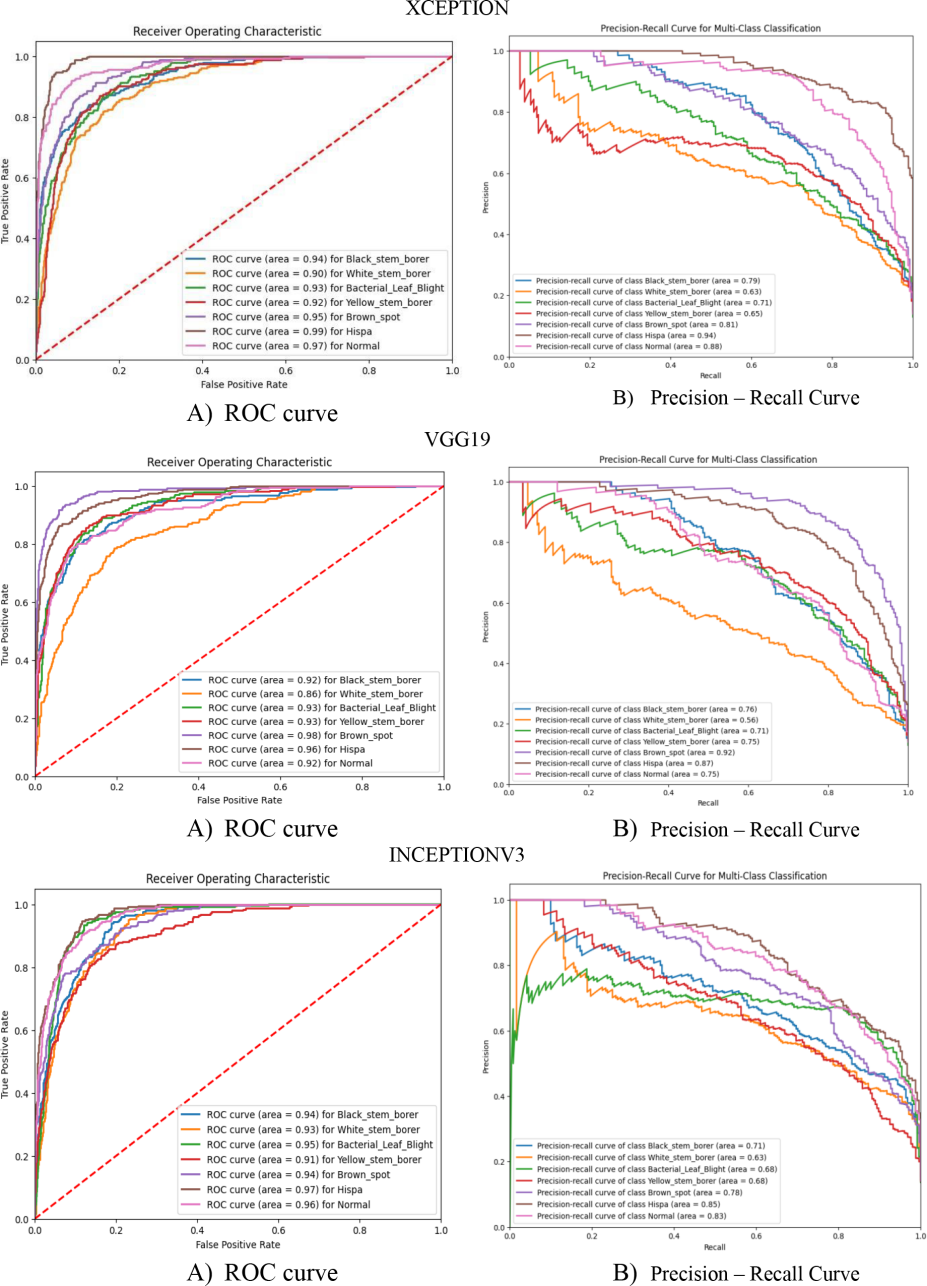
ROC curve and precision–recall of the pre-trained CNN model.

The proposed model’s performance is compared with the other prominent pre-trained CNN models like VGG19, InceptionV3, and Xception. Their individual model accuracy and model loss graph, along with the ROC curve and precision–recall curve, are represented in **Figures 10** and **11**. The performance of the abovementioned CNN pre-trained model is listed in **Tables 8**, **9**, **10**.

**Table 8:** Performance evaluation of VGG19 model.

Classes	Precision	Recall	F1 score	Support	Accuracy
Black_stem_borer	0.61	0.82	0.70	253	74.47%
White_stem_borer	0.72	0.54	0.62	253	
Bacterial_Leaf_Blight	0.64	0.78	0.70	228	
Yellow_stem_borer	0.75	0.81	0.78	259	
Brown_spot	0.96	0.76	0.85	252	
Hispa	0.90	0.81	0.85	268	
Normal	0.74	0.69	0.71	247	

**Table 9:** Performance evaluation of InceptionV3 model.

Classes	precision	Recall	F1 score	Support	Accuracy
Black_stem_borer	0.71	0.78	0.74	251	77.09%
White_stem_borer	0.77	0.76	0.76	244	
Bacterial_Leaf_Blight	0.79	0.89	0.83	240	
Yellow_stem_borer	0.73	0.62	0.67	254	
Brown_spot	0.79	0.73	0.76	275	
Hispa	0.84	0.86	0.82	244	
Normal	0.84	0.77	0.81	249	

**Table 10:** Performance evaluation of Xception model.

Classes	Precision	Recall	F1 score	Support	Accuracy
Black_stem_borer	0.73	0.73	0.73	249	78%
White_stem_borer	0.63	0.78	0.70	251	
Bacterial_Leaf_Blight	0.73	0.66	0.70	229	
Yellow_stem_borer	0.76	0.78	0.77	263	
Brown_spot	0.88	0.74	0.80	253	
Hispa	0.89	0.92	0.90	268	
Normal	0.89	0.83	0.86	247	

Model accuracy and model loss graph for the pre-trained CNN model are shown in **Figure 10**. In this model, accuracy is presented on the left side of the figure and loss on the right side of the figure for Xception, VGG19, and InceptionV3.

ROC and precision–recall graph for the pre-trained model is depicted in **Figure 11** for the Xception, VGG19, and InceptionV3 CNN models. From the precision–recall graph, we can see that the performance of the pre-trained model is not working well for the multiclass dataset chosen.

**Figure 12:**
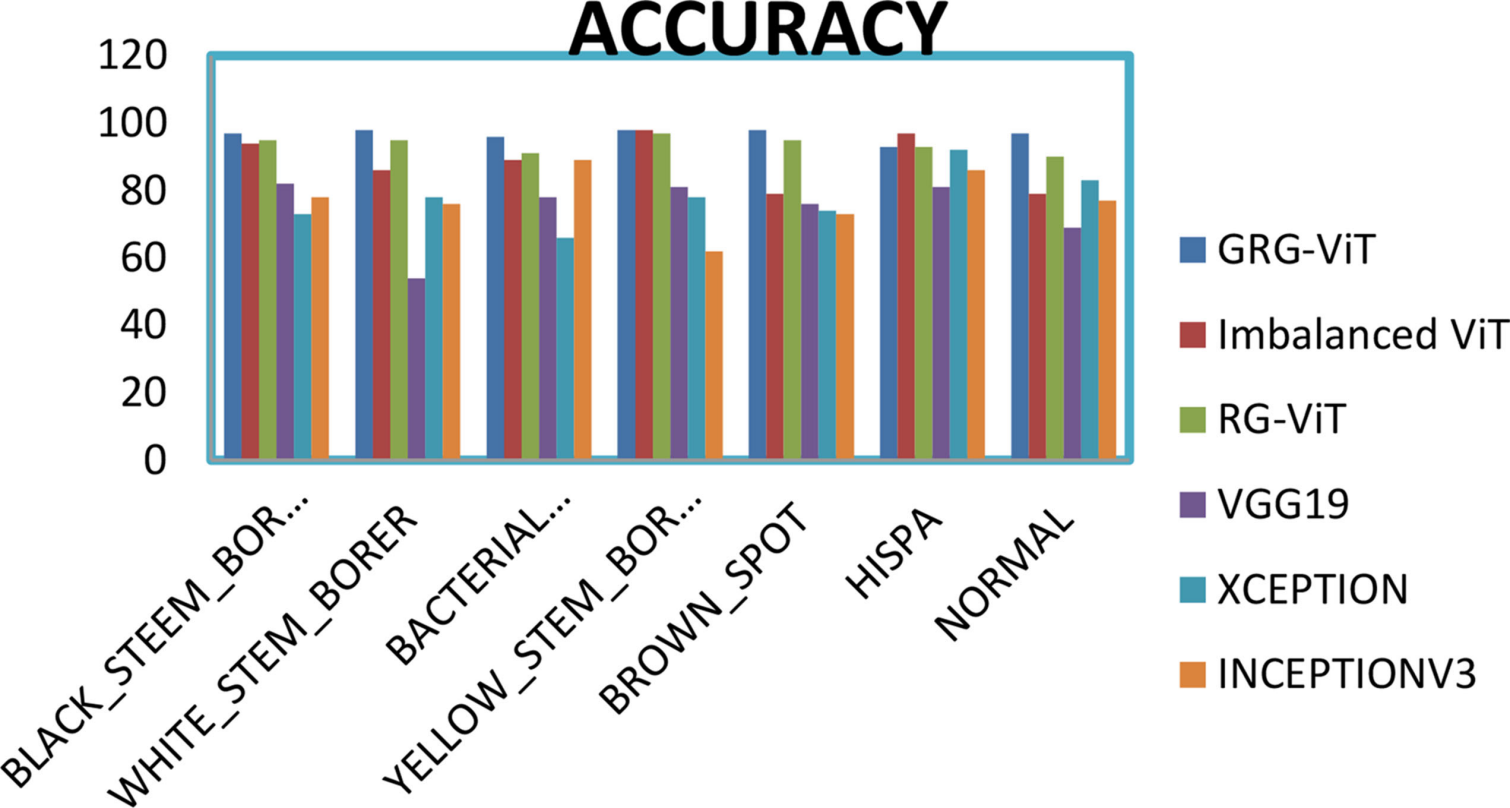
Comparison of the proposed multiclass model with other models.

The individual accuracy of each class for every model is represented in **Figure 12**. From the graph, it is clear that the proposed GRG-ViT model outperforms with an accuracy of 96%.

#### 4.6.1 Confusion matrix

**Figure 13:**
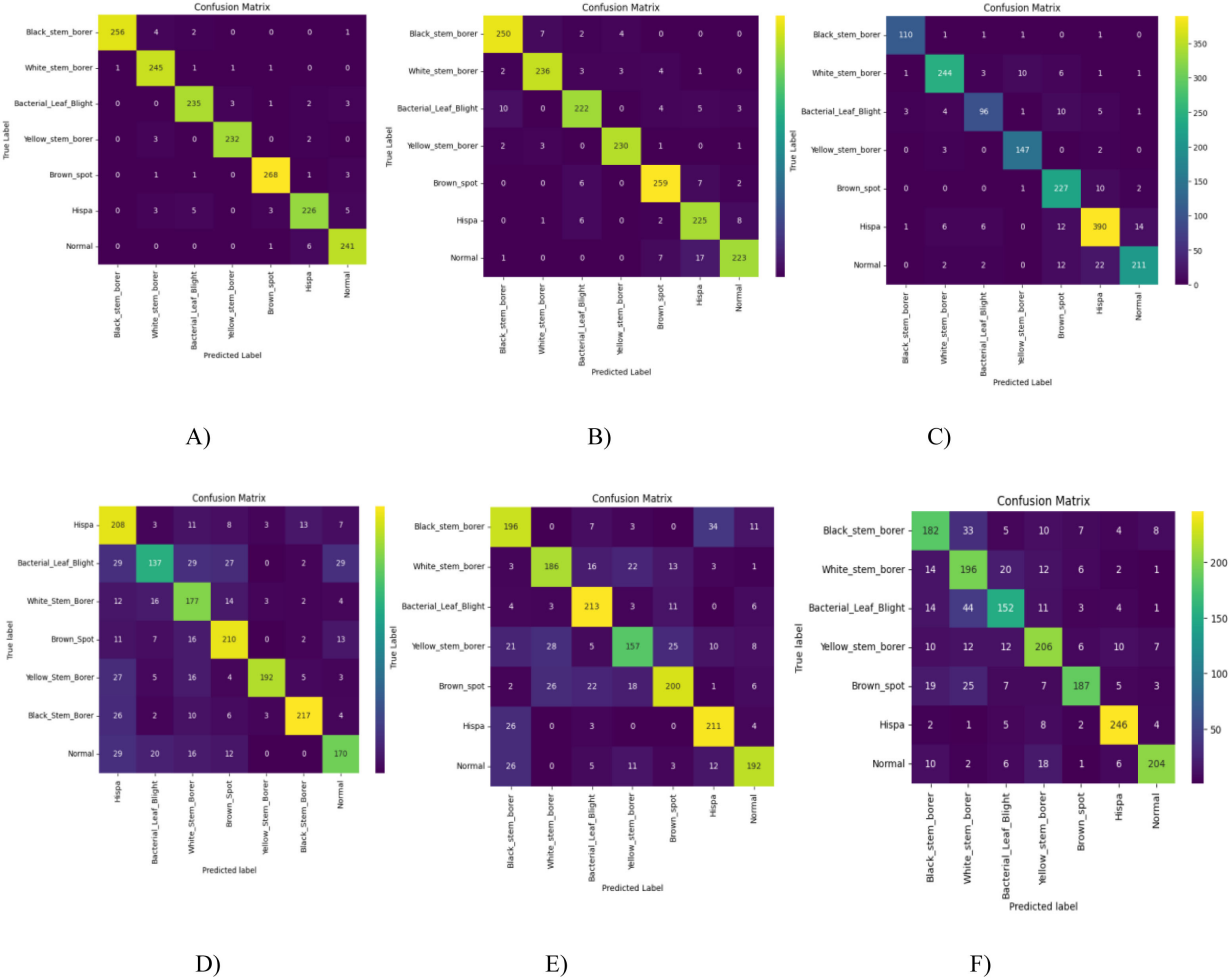
Confusion matrix comparisons between **(A)** GRGViT, **(B)** RGViT, **(C)** ViT with imbalanced dataset, **(D)** VGG19 CNN model, **(E)** InceptionV3 CNN model, and **(F)** Xception CNN model.

To analyze the performance of the model by comparing predicted values with true values, the confusion matrix plays a major role. It evaluates the model by counting true positives, true negatives, false positives, and false negatives. **Figure 13** shows the GRG-ViT model predictions, with true positives and true negatives plotted along the diagonal of the matrix, compared with the ViT model on the imbalanced dataset, the RG-ViT model, and other pre-trained CNN models.

From the detailed presentation of the various results obtained as part of our result analysis, our proposed models have outperformed the other models considered. Detailed outcomes are tabulated and presented in the figures, in terms of ROC measures, confusion matrices, and other important metrics used for model evaluation.

### 4.7 XAI-based interpretation

#### 4.7.1 Grad-CAM mapping

Grad-CAM is one of the most widely used explainable AI techniques for interpreting a model’s performance. It is used in this work to analyze the proposed model’s performance through visualization maps. Grad-CAM highlights the diseased regions of an image, using yellow and red spots for high-intensity regions and blue for low-intensity regions. Yellow spots indicate likely diseased regions, while red spots denote the most important parts of the diseased leaf image, from which the model predicts specific classes. Areas with higher intensity are shown in red using the Grad-CAM heatmap to visualize regions that extract important features for the target class. These two representations are depicted in **Figures 8B and C**, respectively.

#### 4.7.2 Attention visualization map

This Attention Map is specifically introduced for Vision Transformers, where it focuses on the attention mechanism. These maps are generated by aggregating attention weights over different layers and highlight the specific regions of an input image that the model focuses on for making predictions. In the images in **Figure 8**, the attention map highlights the important regions with high intensities, represented using yellow spots surrounded by green, indicating the likely regions. For low-intensity attention areas, blue is used for visualization, as shown in **Figure 8D**.

To validate the model in terms of biologically relevant features, which can help agricultural experts ensure that the proposed model is using appropriate visual cues for classifying different disease classes, these visualization techniques are applied. The sample images of all seven classes are depicted in **Figure 8**.

From **Figure 8**, we can validate the results obtained from our proposed model, and the different XAI approaches illustrate the model’s function. The integration of these techniques helps in observing the working nature of the model, and the figure shows the results for all classes. Thus, this part of our work not only validates the results but also builds trustworthiness in our proposed model.

## 5 Conclusion

Sustainable agricultural productivity is key to ensuring food security, and apart from weather-related threats, diseases cause major damage to productivity. This work presents an advanced deep learning model using Vision Transformer for more accurate disease classification. It addresses class imbalances through GenAI-based techniques, and the results show that the balanced dataset produces better outcomes compared to the imbalanced one. Model interpretability is another key feature of this work, bringing greater reliability to the results, which in turn supports possible real-time deployment of the model. The Grad-CAM and Attention Map visualizations provide evidence-based insights, which not only validate the results but also offer valuable information to agronomists. The key strength of the Vision Transformer is its self-attention mechanism, which naturally captures long-range dependencies. Thus, the proposed model achieves a higher accuracy of 96% compared to other CNN-based models such as VGG19 (74%), InceptionV3 (77%), and Xception (78%). As part of future work, the proposed model will be evaluated in future with the images obtained from the field, which would be captured under varying lighting and background conditions, to assess its robustness and generalization. Furthermore, we also plan to incorporate this proposed model into a lightweight mobile application, which will enable the farmers to recognize the diseases in real time. In addition to this, we will also explore a multimodal architecture to provide a disease-based recommendations tool.

## Data Availability

The original contributions presented in the study are included in the article/supplementary material. Further inquiries can be directed to the corresponding author.
